# COS-7-based model: methodological approach to study John Cunningham virus replication cycle

**DOI:** 10.1186/s12985-018-0939-1

**Published:** 2018-02-05

**Authors:** C. Prezioso, D. Scribano, D. M. Rodio, C. Ambrosi, M. Trancassini, A. T. Palamara, V. Pietropaolo

**Affiliations:** 1 Department of Public Health and Infectous Diseases, Sapienza University of Rome, P.le Aldo Moro, 5, 00185 Rome, Italy; 2 Department of Experimental and Clinical Sciences, “G. D’Annunzio” University of Chieti, Chieti, Italy; 3 Dani Di Giò Foundation–Onlus, Rome, Italy; 4 Department of Public Health and Infectious Diseases, Institute Pasteur, Cenci-Bolognetti Foundation, “Sapienza” University of Rome, Rome, Italy; 5 San Raffaele Pisana Scientific Institute for Research, Hospitalization and Health Care, Rome, Italy

**Keywords:** COS-7 cell line, JCV, Infection, Q-PCR, VP1, Western blot, Immunofluorescence

## Abstract

John Cunningham virus (JCV) is a human neurotropic polyomavirus whose replication in the Central Nervous System (SNC) induces the fatal demyelinating disease, progressive multifocal leukoencephalopathy (PML). JCV propagation and PML investigation have been severely hampered by the lack of an animal model and cell culture systems to propagate JCV have been very limited in their availability and robustness. We previously confirmed that JCV CY strain efficiently replicated in COS-7 cells as demonstrated by the progressive increase of viral load by quantitative PCR (Q-PCR) during the time of transfection and that archetypal regulatory structure was maintained, although two characteristic point mutations were detected during the viral cycle. This short report is an important extension of our previous efforts in defining our reliable model culture system able to support a productive JCV infection.

Supernatants collected from transfected cells have been used to infect freshly seeded COS-7 cell line. An infectious viral progeny was obtained as confirmed by Western blot and immunofluorescence assay. During infection, the archetype regulatory region was conserved.

Importantly, in this study we developed an improved culture system to obtain a large scale production of JC virus in order to study the genetic features, the biology and the pathogenic mechanisms of JC virus that induce PML.

## Introduction

John Cunningham virus (JCV) is the etiological agent of Progressive Multifocal Leukoencephalopathy (PML), a demyelinating disease of the brain [1]. The JCV genome has two conserved protein-coding regions (early and late) transcribed in opposite directions starting from a common regulatory region known as the Non-coding Control Region (NCCR), the most variable portion of the viral genome. It is responsible of tissue tropism, contains the origin of viral DNA replication and promoter/enhancer elements [2]. It controls expression of early and late proteins including large T-antigen (TAg). The DNA sequence of the NCCR distinguishes two forms, the designated archetype and the prototype, which results from a rearrangement of the archetype sequence [3]. Although rearrangement of NCCR of archetype JCV is thought to be an important event in the pathogenesis of PML, little is known regarding what induces this rearrangement [4]. JCV and PML investigation have been severely hampered by the lack of an animal model and the host cell range of archetype JCV is strictly restricted in cultured cells.

Indeed, researchers are focusing their attention to the engineering of several cell lines that support JCV replication and productive infection. The development of models for JCV genome replication is a major breakthrough to understanding viral replication and viral-cell interactions and provides a means to test therapeutic targets. Moreover, there is an urgent need to establish a system to study JCV reactivation and to test drugs directed against JCV migration to the brain. There are several papers describing the ability of JCV to replicate in vitro cell culture models but the efficiency of JCV infection of cultured cells has to be definitively established because it can represent the best system to elucidate JCV behavior inside the host [5–10]. In a previous paper, we produced a model to study the growth characteristics of JCV with archetypal NCCR after transfection and analyzed the NCCR of the progeny viruses for rearrangements that may have occurred [10]. Based on these observations, this short report is addressed to define our reliable model culture system able to support a productive JCV infection.

## Methods

COS-7 cultures, transfection, extraction of viral DNA, Q-PCR, PCR for JCV NCCR and sequencing were previously described [10–16]. COS-7 cells (3.5 × 10^5^) were plated on a 35-mm-diameter dish and cultured overnight in the maintenance medium at 37 °C in the presence of 5% CO_2_. The supernatant, harvested from previous transfection experiments with JCV CY strain DNA, was subjected to 6 cycles of freezing and thawing, followed by centrifugation at 2000 rpm for 5 min. The resulting clarified supernatant, containing virions corresponding to 3.38 × 10^5^ genome equivalents per milliliter (gEq/ml), was used to infect freshly seeded COS-7 cells.

After adsorption for 2 h, the cells were washed 3 times with PBS and incubated with fresh medium for 4 days and then transferred into 90-mm-diameter dishes. Subsequently, the cells were continuously cultured in the maintenance medium and split at 1:5 ratio every 7 days. Cells and supernatants were collected once a week until 35 days post-infection (d.p.i.). Viral DNA was extracted from 1 × 10^6^ COS-7 cells. DNA extracted and relative supernatants were quantified using Q-PCR for TAg. The data were expressed as genome equivalents (gEq) of viral DNA per cell DNA content (gEq/cell DNA content) for COS-7 cells and as genome equivalents (gEq) of viral DNA per milliliter (gEq/ml) for supernatants. COS-7 cells and supernatants, collected one time a week until 35 d.p.i., were used for Western blot analysis. The concentration of proteins in each supernatant was determined using the BCA protein assay kit (Pierce BCA Protein Assay Kit, Thermo Scientific, Waltham, MA, USA). Equal amounts of protein from supernatants and whole cell protein extracts were separated by sodium dodecyl sulphate-polyacrylamide gel electrophoresis (SDS-PAGE), transferred to polyvinylidene fluoride (PVDF) membranes (Bio-Rad) and blocked with non-fat dry milk 5% (NFDM, Bio-Rad). The membranes were incubated with the mouse monoclonal anti-human polyomavirus JCV capsid protein VP1 antibody (1:400 dilution; ab34756 Ab-cam) and with the anti-mouse HRP-conjugated antibody (Bio-Rad). To normalize the total protein content in COS-7 cells, blots were probed for glyceraldehyde-3-phosphate dehydrogenase (GAPDH) using the anti-GAPDH antibody (1:8000 dilution; ab9485 Ab-cam). The signals were detected with enhanced chemi-luminescence reagents (GE, Healthcare). Band intensities were quantified by densitometry analysis using the ImageJ software [17]. Immunofluorescence (IF) was used to detect VP1 intracellular localization. Infected and uninfected COS-7 cell monolayers were fixed with 4% paraformaldehyde and permeabilized for 10′ with a 0.3% solution of Triton X-100 in PBS. Cell monolayers were incubated with a mouse monoclonal anti-human polyomavirus JC capsid protein VP1 primary antibody (1:250 dilution; ab34756 Ab-cam) and with Alexa Fluor 568 goat anti-mouse secondary antibody (1:1000 dilution; A11004 Invitrogen). Cells were washed with PBS and cellular DNAs were labeled with 4′, 6′-diamidino-2-phenylindole (DAPI, Molecular Probes). Single images were acquired with a Leica DM5000B microscope equipped with the Digital FireWire Color and Black and White Camera systems LeicaDFX350 and DFX300, respectively, and processed using the Leica Application Suite 2.7.0.R1 software (Leica). Data obtained were summarized as medians and ranges or as mean ± standard deviation, as appropriate. Data were analyzed by Student’s t-test. *P* < 0.05 were considered statistically significant.

## Results and discussion

Our experimental workflow started from previous results obtained from transfection experiments of COS-7 cells with the genome DNA from JCV CY strain. We showed a JCV efficiently replication in COS-7 cells as demonstrated by the progressive increase of viral load during the time of transfection [10]. From this basis, we wanted to go further in developing an infection model reviewing the physiological viral life-cycle in order to consolidate that this cell model can support an efficient archetype JCV replication and can produce an infectious progeny.

Hence, a known amount of virions, corresponding to 3.38x10^5^gEq/ml and collected from previous transfection experiment, was used to infect freshly seeded COS-7 cells.

To start with, cells and supernatants were collected and harvested one time a week until 35 d.p.i. from 4 d.p.i. Intracellular viral DNA was extracted from 1 × 10^6^ COS-7 cells after infection at the selected sampling time and used in Q-PCR for JCV TAg in order to evaluate the efficiency of JCV replication. Supernatants were directly used in molecular biology assays.

**Fig. 1:**
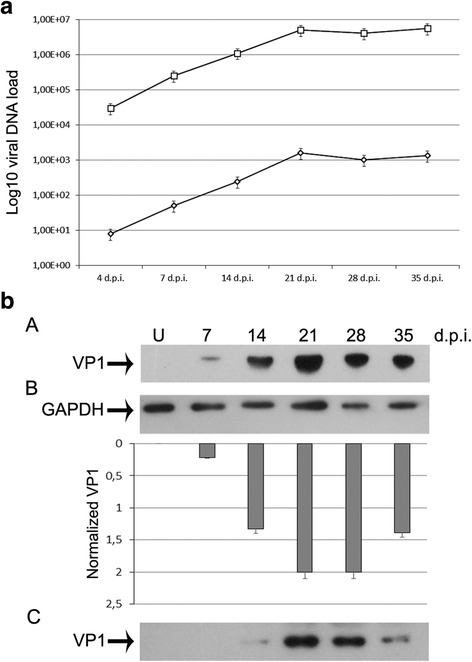
**a** Replication studies of archetype JCV in COS-7 cells and in supernatants during infection. The efficiency of JCV replication during infection was assessed at selected sampling times in cells and in the supernatants collected one time a week from 4 days post infection (d.p.i.) and after 7 d.p.i., 14 d.p.i., 21 d.p.i., 28 d.p.i. until 35 d.p.i. Extra and intracellular JCV DNAs were quantified by Q-PCR of supernatants and extracted DNA from cells respectively, harvested at the indicated time points. The increase of JCV replication in COS-7 cells (diamonds) and in the supernatant (squares) during the infection experiments is shown. Data were expressed as the mean of three independent experiments, error bars represented standard deviations. For COS-7 cells data were expressed as genome equivalents (gEq) of viral DNA per cell DNA content (gEq/cell DNA content) and for supernatants as genome equivalents (gEq) of viral DNA per milliliter (gEq/ml). **b** Analysis of VP1 expression in COS-7 cells and in supernatants during infection. Cell protein extracts (Panel A) and supernatants (Panel C) from uninfected (U) and infected COS-7 cells were harvested at 7 d.p.i., 14 d.p.i., 21 d.p.i., 28 d.p.i. and 35 d.p.i. and analyzed by Western blot to evaluate the expression of the VP1 protein. Equal amounts of protein from supernatants and whole cell protein extracts were separated by SDS-PAGE, transferred to PVDF and probed using anti-VP1 antibody. Bars depict the expression level of VP1 protein during infection quantified by densitometry (ImageJ software), and normalized to host cell number (VP1/GAPDH) (Panel B). Data are expressed as arbitrary units and are means ± SD from at least three independent experiments

Results of Q-PCR obtained from three independent experiments revealed a progressive increase of viral replication in cells and in supernatants. In fact, at 4 d.p.i. a JCV viral load average of 4.96x10^1^gEq/cell DNA content was detected, reaching the maximum value of 1.60x10^3^gEq/cell DNA content at 35 d.p.i. (Fig. 1a). In parallel, JCV replication evaluated in the supernatants showed the same trend to that already observed in the cells. The value of viral DNA progressively increased, with the highest value of viral DNA detected 35 d.p.i. (average value of 5.59x10^6^gEq/ml) (Fig. 1a).

In order to test whether an infectious progeny has been produced during infection in COS-7 cells, total protein extracts from COS-7 cells were used to assess capsid protein VP1 production using Western blot analysis. Interestingly, within COS-7 cells Western blot showed that VP1 protein as early as expressed 7 d.p.i. and its level increased during the infection experiments, reaching the maximum expression at 21 d.p.i. (Fig. 1b panel A). To further corroborate our results, we not only performed Western blot analysis for VP1 expression in infected cells, but also monitored VP1 protein in the supernatants. As shown in Fig. 1b panel C, VP1 expression was detected 14 d.p.i. within supernatants as consequence of the release of final viral products via host cell lysis.

Moreover, VP1 protein was detectable in the cells and in the supernatants up to 35 d.p.i. (Fig. 1b panel A and panel C). These results are indicative of the presence of infectious viral particles both in cells and in supernatants, demonstrating that a viral infectious progeny is assembled in COS-7 cell line and then released. VP1 protein is responsible for the icosahedral structure of JCV and contains the epitopes for antibody induction and recognition. It represents the most important viral protein playing a role in JCV pathogenesis, in mediating the immune response, in host cell attachment and in viral cell entry [18].

**Fig. 2:**
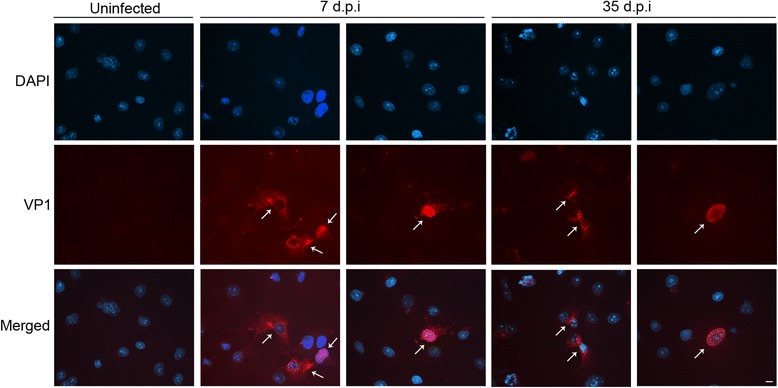
Intracellular VP1 localization during infection. COS-7 cells were infected with JCV-containing supernatants (3.38 × 10^5^ gEq/ml) for 2 h at 37 °C in the presence of 5% CO_2_, after medium replacement the cells were cultured for 35 days for the time course infection experiment. At selected time points cells were fixed and stained with anti-human polyomavirus JCV capsid protein VP1 and the anti-mouse Alexa Fluor 568 conjugated (Red). The cell nuclei (blue) were stained with DAPI. Images, representatives of three independent experiments, were acquired using a Leica camera and processed using Qwin software (Leica). Arrows point VP1 localization into the host cell cytoplasm and/or into the nucleus. Uninfected cells were used as control. Scale bar, 10 μm

Finally, the intracellular localization of JCV VP1 expressed in COS-7 cells was monitored by IF experiments. As expected, JCV VP1 was detected both in cell cytoplasm and in the nucleus (Fig. 2). This localization was observed in all time course infection experiment, starting from 7 d.p.i. until 35 d.p.i. (Fig. 2). VP1 was efficiently transported into the nucleus for viral assembly to form the complete virion particles. The mechanism of VP1 transport through the cytoplasm towards the nucleus is due to the presence of the nuclear localization signal of VP1 (MAPT K5R6K7 GEK8K9D) in its N-terminus sequence, as described in previously reports present in literature [19, 20].

Q-PCR, Western Blot and IF results suggest that JC virus was able to replicate its DNA, express VP1 and produce infectious progeny that was capable of reinfecting COS-7 cells.

This improved culture system for archetype JCV is more adapt than other COS-7 model present in literature, since it supplants previous models that have not demonstrated a production of infectious progeny in COS-7cell line.

Our model could be used to try to study one of the unresolved question regarding the ability of archetype JCV NCCR to mutate. In fact the NCCR is the highest variable sequence in the JCV genome. The mechanisms, through which JCV rearranges and generates virions able to reach the brain and to induce PML, are not understood yet [21, 22]. Hence, the study of cell lines that support the process of JCV rearrangement is fundamental to predict PML development. This is the missing piece in the pathogenesis puzzle of PML. The importance of NCCR rearrangements in the onset of PML has been demonstrated by the discovery of highly rearranged sequences in patients with this condition. However, it is not yet understood where and when these rearrangements occur, if during primary infection or during the persistence of the virus inside the host [23, 24]. For all the reason, an important novelty of our work was the analysis, in time course infection experiments, of the archetypal NCCR to identify mutations that can occurs during viral replication and to study if the rate and type of mutations are related to the cell lines used. During infection, the sequence analysis of JCV NCCR showed an archetype CY-like structural organization. Some point mutations were found in the different sequenced samples but they did not involve known binding sites for transcriptional factors (data not shown). We focused on mutations within the binding sites for host transcriptional factors since they are involved in viral transcription and control of viral tropism. In particular, the 37 T to G nucleotide transversion within the binding site for the cellular transcription factor Spi-B in box B and the 217 G to A nucleotide transition in box F at the level of the binding site for the cellular transcription factor NF-1 already observed during transfection were maintained [10]. These two point mutations of putative clinical significance, previously reported in urine of immunocompetent individuals [25] and in urine and PBMC of multiple sclerosis patients under Natalizumab treatment [26], were detected for the first time in an in vitro system and were sign of an active replication of the viral DNA.

## Conclusion

There are many gray areas in our understanding of JCV biology therefore scientific literature motivate the effort to develop in vitro models to study the molecular bases of latency and reactivation, the site(s) of persistency, the relationship of archetype and prototype virus. Importantly, in this study we developed an improved culture system to obtain a large scale production of archetype JC virus. This could represent the best approach to collect infectious viral progeny to be used in studying of JCV behavior inside host cell and to test drugs directed against JCV. In fact, to date, no effective antiviral therapy is presently available to treat patients with PML. More in general, this study synthesizes a methodological approach that could be helpful to define if archetype JCV might be converted to PML-type virus during the replication in the site of viral persistent and to define NCCR rearrangements as possible viral biomarkers for an early PML diagnosis.

## Abbreviations

217 G → A: 217 G to A nucleotide transition
37 T → G: 37 T to G nucleotide transversion
CNS: Central Nervous System
d.p.i.: Days post infection
DAPI: 4′,6-diamidino-2-phenylindole
GAPDH: Glyceraldehyde-3-phosphate dehydrogenase
gEq: Genome equivalents
gEq/cell DNA content: Viral DNA per cell DNA content
gEq/ml: Genome equivalents per milliliter
IF: Immunofluorescence
JCV: John Cunningham virus
NCCR: Non-coding control region
NFDM: Non-fat dry milk
PML: Progressive multifocal leukoencephalopathy
PVDF: Polyvinylidene fluoride
Q-PCR: Quantitative PCR
SDS-PAGE: Sodium dodecyl sulphate-polyacrylamide gel electrophoresis
TAg: Large T antigen

## References

[1] Padgett BL, Walker DL, Zu Rhein GM, Echroade RJ, Dessel BH (1971). Cultivation of papova-like virus from human brain with progressive multifocal leukoencephalopathy. Lancet.

[2] Frisque RJ, Bream GL, Cannella MT (1984). Human polyomavirus JC virus genome. J Virol.

[3] Marshall LJ, Major EO (2010). Molecular regulation of JC virus tropism: insights into potential therapeutic targets for progressive multifocal leukoencephalopathy. J NeuroImmune Pharmacol.

[4] Major EO, Amemiya K, Tornatore CS, Houff SA, Berger JR (1992). Pathogenesis and molecular biology of progressive multifocal leukoencephalopathy, the JC virus-induced demyelinating disease of the human brain. Clin Microbiol Rev.

[5] Hara K, Sugimoto C, Kitamura T, Aoki N, Taguchi F, Yogo Y (1998). Archetype JC virus efficiently replicates in COS-7 cells, simian cells constitutively expressing simian virus 40 T antigen. J Virol.

[6] Nukuzuma S, Kameoka M, Sugiura S, Nakamichi K, Nukuzuma C, Miyoshi I, Takegami T (2009). Archetype JC virus efficiently propagates in kidney-derived cells stably expressing HIV-1 tat. Microbiol Immunol.

[7] Randhawa P, Zeng G, Bueno M, Salgarkar A, Lesniak A, Isse K, Seyb K, Perry A, Charles I, Hustus C, Huang M, Smith M, Glicksman MA (2014). Inhibition of large T antigen ATPase activity as a potential strategy to develop anti-polyomavirus JC drugs. Antivir Res.

[8] Broekema NM, Imperiale MJ (2012). Efficient propagation of archetype BK and JC polyomaviruses. Virology.

[9] Major EO, Miller AE, Mourrain P, Traub RG, de Widt E, Sever J (1985). Establishment of a line of human fetal glial cells that supports JC virus multiplication. Proc Natl Acad Sci U S A.

[10] Prezioso C, Scribano D, Bellizzi A, Anzivino E, Rodio DM, Trancassini M, Palamara MT, Pietropaolo V (2017). Efficient propagation of archetype JC polyomavirus in COS-7 cells: evaluation of rearrangements within the NCCR structural organization after transfection. Arch Virol.

[11] Delbue S, Branchetti E, Boldorini R, Vago L, Zerbi P, Veggiani C, Tremolada S, Ferrante P (2008). Presence and expression of JCV early gene large T antigen in the brains of immunocompromised and immunocompetent individuals. J Med Virol.

[12] Pietropaolo V, Videtta M, Fioriti D, Mischitelli M, Arancio A, Orsi N, Degener AM (2003). Rearrangement patterns of JC virus noncoding control region from different biological samples. J Neuro-Oncol.

[13] Flaegstad T, Sundsfjord A, Arthur RR, Pedersen M, Traavik T, Subramani S (1991). Amplification and sequencing of the control regions of BK and JC virus from human urine by polymerase chain reaction. Virology.

[14] Markowitz RB, Thompson HC, Mueller JF, Cohen JA, Dynan WS (1993). Incidence of BK virus and JC virus viruria in human immunodeficiency virus infected and uninfected subjects. J Infect Dis.

[15] Kwok S, Higuchi R (1989). Avoiding false positive with PCR. Nature.

[16] ClustalW2 - mu5ltiple sequence alignment. http://www.ebi.ac.uk/Tools/ msa/clustalw2/. Accessed 25 Jan 2018.

[17] Rasband WS, ImageJ, U. S. National Institutes of Health, Bethesda, Maryland, USA, 1997-2016, https://imagej.nih.gov/ij/. Accessed 25 Jan 2018.

[18] Weissert R (2011). Progressive multifocal leukoencephalopathy. J Neuroimmunol.

[19] Shishido-Hara Y, Hara Y, Larson T, Yasui K, Nagashima K, Stoner GL (2000). Analysis of capsid formation of human polyomavirus JC (Tokyo-1 strain) by a eukaryotic expression system: splicing of late RNAs, translation and nuclear transport of major capsid protein VP1, and capsid assembly. J Virol.

[20] Saribas AS, DeVoto J, Golla A, Wollebo HS, White MK, Safak M. Discovery and characterization of novel trans-spliced products of human polyoma JC virus late transcripts from PML patients. J Cell Physiol. 2017:1–19. 10.1002/jcp.26219. 10.1002/jcp.26219 PMC5805571 29044559

[21] White MK, Khalili K (2011). Pathogenesis of progressive multifocal leukoencephalopathy—revisited. J Infect Dis.

[22] Major EO (2010). Progressive multifocal leukoencephalopathy in patients on immunomodulatory therapies. Ann Rev Med.

[23] Chapagain ML, Nerurkar VR (2010). Human polyomavirus JC (JCV) infection of human B lymphocytes: a possible mechanism for JCV transmigration across the blood–brain barrier. J Infect Dis.

[24] Wollebo HS, White MK, Gordon J, Berger JR, Khalili K (2015). Persistence and pathogenesis of the neurotropic polyomavirus JC. Ann Neurol.

[25] Agostini HT, Ryschkewitsch CF, Stoner GL (1996). Genotype profile of human polyomavirus JC excreted in urine of immunocompetent individuals. J Clin Microbiol.

[26] Pietropaolo V, Bellizzi A, Anzivino E, Iannetta M, Zingaropoli MA, Rodio DM, Morreale M, Pontecorvo S, Francia A, Vullo V, Palamara AT, Ciardi MR (2015). Human polyomavirus JC replication and non-coding control region analysis in multiple sclerosis patients under natalizumab treatment. J Neuro-Oncol.

